# Learning about loss

**DOI:** 10.7554/eLife.00533

**Published:** 2013-09-10

**Authors:** Alejandro Sánchez Alvarado

**Affiliations:** 1 **Alejandro Sánchez Alvarado** is an *eLife* reviewing editor, and is at the Howard Hughes Medical Institute, Stowers Institute for Medical Research, Kansas City, Missouri, United Statesasa@stowers.org

## Abstract

Researchers have identified two genes—*follistatin* and *activin*—as having an important role in the ability of certain flatworms to identify wounds that require the production of new tissue.

**Related research article** Gaviño MA, Wenemoser D, Wang IE, Reddien PW. 2013. Tissue absence initiates regeneration through Follistatin-mediated inhibition of Activin signaling. *eLife* **2**:e00247. doi: 10.7554/eLife.00247

**Image** A flatworm in which neoblasts—stem cells that can give rise to multiple cell types—have been labelled

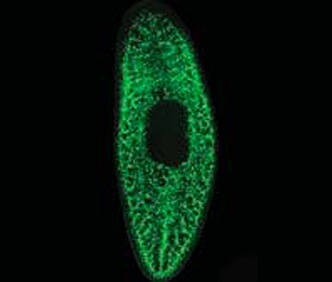

Some animals show remarkable powers of regeneration. A salamander that loses a limb can grow a replacement, while a zebrafish can replace a damaged heart. And a planarian—a type of flatworm—can re-grow an entire head following decapitation. But how does such regeneration occur in these animals? And why are mammals and many other species, both vertebrates and invertebrates, found wanting in this regard? The problem is particularly vexing when one considers that regeneration is triggered by wounding; why is it that most, if not all, animals can heal their wounds, but only a few can regenerate body parts lost to such injuries? These questions have puzzled thinkers, naturalists and scientists for millennia. Over the past decade, however, it has become possible both to explore the influence of specific genes on the mechanisms that underpin regeneration, and to begin to answer these long-standing questions (Figure 1; Sánchez Alvarado and Tsonis, 2006; Tanaka and Reddien, 2011).

**Figure 1.**
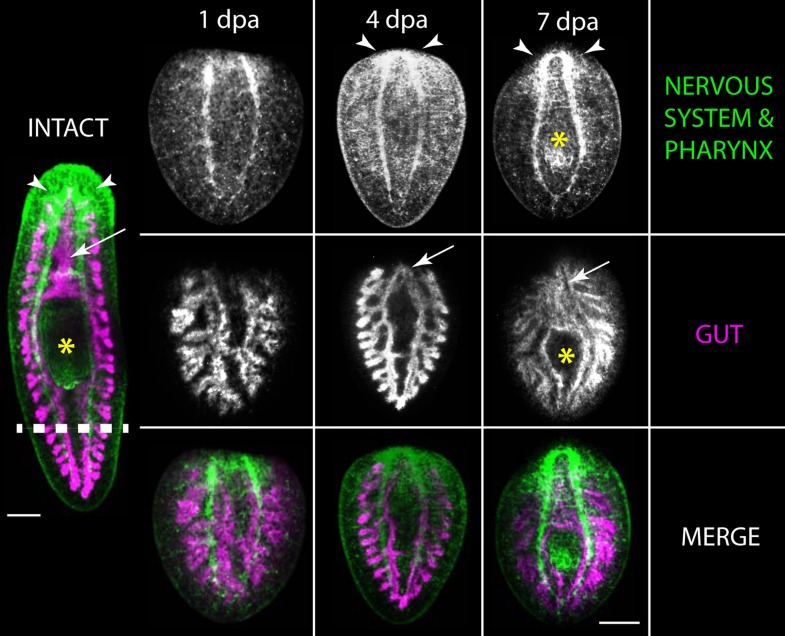
Upon injury, planarians can regenerate lost tissues. An intact, live planarian (left) had its tail amputated (dashed line). The tail fragment regenerated into a new planarian, shown at 1, 4 and 7 days post-amputation (dpa). The regenerating tail is labelled to show the nervous system and pharynx (also known as the feeding organ; yellow asterisk) (top) and the gut (middle), while the lower panel shows a merged image with the nervous system and pharynx in green, and the gut in purple. Scale bar: 200 μm. Adapted from Elliot et al. (2012).

Part of the challenge is to distinguish between wounds that involve tissue loss and therefore require regeneration, and those wounds for which a simple repair will be sufficient. Now, writing in *eLife*, Michael Gaviño, Danielle Wenemoser, Irving Wang and Peter Reddien, all at the Massachusetts Institute of Technology, provide fresh insights into the ways in which planarians—a key model organism used in studies of regeneration (Elliott and Sánchez Alvarado, 2012)—may distinguish between these two scenarios (Gaviño et al., 2013).

Given that regeneration requires extensive coordination of events at both the molecular and cellular level, it is logical to expect that cell–to–cell communication will have a central role (Sánchez Alvarado, 2004). Over the past 30 years, a number of highly conserved signalling pathways have been identified in embryonic development and, more recently, researchers have investigated whether these pathways also contribute to adult developmental processes, such as regeneration. The Wnt-signalling pathway, for example, has long been known for its multiple roles in embryogenesis. Moreover, this pathway is now also thought to be implicated in the regulation of wound responses and regenerative mechanisms across the animal kingdom, from simple animals such as Hydra (Hobmayer et al., 2000) to more complex organisms such as planarians (Gurley et al., 2008; Petersen and Reddien, 2008), zebrafish (Stoick-Cooper et al., 2007) and mammals (Clevers and Nusse, 2012).

When Gaviño and colleagues systematically examined the genes that are expressed in planarians in response to wounding, they identified an early and sustained activation of genes that regulate another signalling pathway, known as the TGF-β pathway. To date, this pathway has been implicated primarily in patterning the dorsoventral axis during development (that is, establishing the ‘front’ and ‘back’ of animals) (Petersen and Reddien, 2008; Moustakas and Heldin, 2009). Wounded planarians displayed increased expression of genes called *activin-2* and *follistatin.* Activin is a protein complex that binds to a receptor to activate the TGF-β signalling cascade, whereas Follistatin is an inhibitor protein that prevents Activin from binding to its receptor.

Gaviño, Wenemoser, Wang and Reddien then used a technique known as RNA-mediated genetic interference (RNAi) to inhibit the *follistatin* and *activin* genes and observed the response of these planarians to injury. In planarians that had experienced amputation, the inhibition of *follistatin*—which leaves *activin* free to activate the TGF-β signalling pathway—blocked key cellular and tissue remodelling events associated with tissue loss, and prevented regeneration. Interestingly, this is the opposite of what has been reported for vertebrates, in which TGF-β signalling promotes rather than suppresses regeneration.

Conversely, inhibition of the *activin-1* gene resulted in increased regenerative progenitor formation. Nevertheless, inhibition of *activin-1* alone is not sufficient to activate wound-induced gene expression in planarians that have not been injured. It is therefore likely that the TGF-β signalling pathway is not the only player involved in the decision to mount a regenerative response.

The lack of regeneration in planarians in which RNAi has been used to inhibit the *follistatin* gene suggests that the Follistatin protein may serve as a biosensor that gauges the extent of tissue loss. Consistent with this hypothesis, Gaviño and colleagues observed that *follistatin* is expressed for longer times at wounds with tissue loss than at those without, and that expression levels correlate with the amount of tissue that has been removed. These observations support a model in which injury induces both *follistatin* and *activin* expression, with *follistatin* expression regulated by the degree of tissue loss. In this model, *activin* inhibits the regenerative response; after a major injury, however, high levels of *follistatin* overcome the effects of Activin and promote regeneration. The behaviour of these genes provides an interesting paradigm in which to study the regulation of promoters in general and, in particular, to explore how the seemingly quantitative regulation of the *follistatin* promoter in planarians is related to their responses when they are wounded.

Nevertheless, the agents that mediate the activation of *follistatin* and *activin* gene expression remain to be determined. In fact, a recent article by Roberts-Galbraith and Newmark reports that *follistatin* acts in combination with *notum* (an antagonist of the Wnt pathway) to reestablish a signalling center that may direct cells towards specific fates (Roberts-Galbraith and Newmark, 2013). Moreover, there are likely to be other as yet unidentified ‘missing tissue’ signals that are produced in response to amputation. The fact that stem cells still migrate to sites of injury in planarians in which *follistatin* has been targeted via RNAi clearly supports the existence of regenerative factors that are independent of the Follistatin-mediated inhibition of Activin. I suspect that the identification and characterization of these agents will keep biologists busy for the foreseeable future. Such factors, whatever their nature, will inform us about the processes unleashed by wounding that elicit healing and/or regeneration. Their identification will also shed light on why some organisms are much more adroit at regeneration than others. This, in turn, may help identify strategies to extend regenerative powers to tissues of medical importance that lacking in this property.
