# Correction: Learning about loss

**DOI:** 10.7554/eLife.01597

**Published:** 2013-10-08

**Authors:** Alejandro Sánchez Alvarado

Sánchez Alvarado A. 2013. Learning about loss. *eLife* **2**:e00533. doi: 10.7554/eLife.00533. Published 10 September 2013

An error was introduced into the fourth paragraph of this article by eLife editorial. The third sentence of this paragraph originally referred to *activin-1* where it should have referred to *activin-2*.

The article has been corrected accordingly.

